# Tunneling Nanotube: An Enticing Cell–Cell Communication in the Nervous System

**DOI:** 10.3390/biology12101288

**Published:** 2023-09-27

**Authors:** Sunayana Dagar, Srinivasa Subramaniam

**Affiliations:** 1 Department of Neuroscience, The Herbert Wertheim UF Scripps Institute for Biomedical Innovation & Technology, Jupiter, FL 33458, USA; 2 The Scripps Research Institute, La Jolla, CA 92037, USA; 3 Norman Fixel Institute for Neurological Diseases, 130 Scripps Way, C323, Jupiter, FL 33458, USA

**Keywords:** tunneling nanotubes, Rhes, neurodegenerative diseases, mutant huntingtin, striatum, cargo transmission, dopamine receptors

## Abstract

**Simple Summary:**

This review highlights the role of tunneling nanotubes (TNTs) and TNT-like structures in the nervous system. TNTs are thin, hollow plasma membrane projections that directly connect the lumen of one cell to the lumen of another cell, thereby transferring different cargoes between the two connected cells. TNTs have been shown to play very prominent roles in neuronal development and serve as highways for neurodegenerative diseases in the brain. Here, we discuss different crucial aspects of TNTs like their architecture, TNTs’ involvement in different pathological and physiological conditions in the nervous system, and the mechanisms involved in their formation. In this review, we also discuss different challenges involved in TNT research and its future prospects.

**Abstract:**

The field of neuroscience is rapidly progressing, continuously uncovering new insights and discoveries. Among the areas that have shown immense potential in research, tunneling nanotubes (TNTs) have emerged as a promising subject of study. These minute structures act as conduits for the transfer of cellular materials between cells, representing a mechanism of communication that holds great significance. In particular, the interplay facilitated by TNTs among various cell types within the brain, including neurons, astrocytes, oligodendrocytes, glial cells, and microglia, can be essential for the normal development and optimal functioning of this complex organ. The involvement of TNTs in neurodegenerative disorders, such as Alzheimer’s disease, Huntington’s disease, and Parkinson’s disease, has attracted significant attention. These disorders are characterized by the progressive degeneration of neurons and the subsequent decline in brain function. Studies have predicted that TNTs likely play critical roles in the propagation and spread of pathological factors, contributing to the advancement of these diseases. Thus, there is a growing interest in understanding the precise functions and mechanisms of TNTs within the nervous system. This review article, based on our recent work on Rhes-mediated TNTs, aims to explore the functions of TNTs within the brain and investigate their implications for neurodegenerative diseases. Using the knowledge gained from studying TNTs could offer novel opportunities for designing targeted treatments that can stop the progression of neurodegenerative disorders.

## 1. Introduction

Intercellular communication in the nervous system plays a critical role in coordinating essential functions. TNTs have recently emerged as fascinating avenues of intercellular communication [1,2,3,4]. TNTs are membranous cell–cell communication structures that were first reported in neuronal phaeochromocytoma 12 (PC12) cells [5]. They are known to establish connections between various cell types within the nervous system, including neurons, astrocytes, microglia, and pericytes [6,7,8,9,10,11,12,13]. By facilitating the direct exchange of vital information and cargo, TNTs can contribute to the regulation of neuronal activity, synaptic plasticity, and neuroinflammatory responses [14,15,16,17]. Furthermore, they can play crucial roles in the development and maintenance of neural circuits [15,18].

TNTs have received much attention due to their connection to neurodegenerative diseases. Studies have implicated TNTs in diseases such as Alzheimer’s disease (AD), Creutzfeldt–Jakob disease, Huntington’s disease (HD), and Parkinson’s disease (PD) [3,4,11,19,20,21,22,23,24]. The spread of disease-causing pathological proteins between neurons through TNTs can contribute to the propagation of disease across different brain areas [21,25]. Moreover, TNTs can facilitate the transmission of neurotoxic factors and detrimental signals like amyloid-β, reactive oxygen species (ROS), cholera toxin B, and apoptotic signals, exacerbating the progression of these disorders [7,26].

TNTs have also been implicated in brain cancer, particularly glioblastoma [27,28,29,30]. Here, TNT-like communication plays a role in disease progression by supporting microenvironmental conditions that promote cancer cell survival and proliferation. Through TNTs, tumor cells can transfer essential cellular components, including growth factors and genetic material, which enhance their resistance to treatment [27]. Thus, targeting TNT-mediated intercellular communication may represent a potential strategy to disrupt tumor-supportive networks and improve the efficacy of cancer therapies.

Despite the growing recognition of TNTs’ significance in the nervous system and brain, there are technical challenges associated with their identification and visualization in vivo and within complex brain tissues. However, new developments in imaging methods, including super-resolution microscopy, along with the application of organoid models, have offered insightful knowledge on TNT biology in a more physiologically relevant setting [31,32,33,34]. These advancements pave the way for further exploration of TNT function and dynamics within complex neural networks.

This review article, based on our recent works on TNTs [2,3,4,22,23,35], will concentrate on studies examining the role of TNTs in the nervous system and their involvement in neurodegenerative diseases. Neurons, which are distinct from other cells, possess specialized abilities for long-range communication through electrical and chemical signals. Enhancing our comprehension of the molecular processes that govern neural TNT activity and investigating its therapeutic prospects, as well as devising novel approaches to manipulate TNT function, may have profound consequences for the management of neurological diseases and brain malignancies.

## 2. TNT Architecture

TNTs, characterized by their thin, long, and flexible membranous structure, allow for the transfer of organelles, vesicles, and proteins between cells [1,2,15]. Unlike other cellular components, TNTs lack specific markers for identification, and thus, their morphological features, such as their diameter and length, and their ability to grow above the substratum in 2D culture, serve as the primary criteria for recognition. Based on diameter, TNTs can be loosely classified into two groups: those with a diameter of less than 0.7 µm, which are primarily composed of actin and carry small membranous cargoes, and those with a diameter larger than 0.7 µm, which contain both actin and microtubules and are capable of transporting larger cytoplasmic components [36]. The post-translational modifications (PTMs) like acetylation and detyrosination on tubulin in swine testicle cells have been shown to maintain the stability of tunneling nanotubes (TNTs) over longer distances [37]. In 2D culture, TNTs can be observed as structures that appear to “hang” between the connected cells. They are often transient in nature and exhibit varying lifetimes. For example, in neuronal cells, TNTs can last for minutes [38,39], while in normal rat kidney cells, they can persist for several hours [40,41]. While there is still much to be learned, it is generally understood that TNTs can be generated by two distinct mechanisms, growth and extension, like filopodia, or through the gradual detachment of adhering cells while preserving filamentous connections. TNTs can establish closed-ended or open-ended connections with target cells, but the mechanism for such selections remains unknown [2,14].

A recent study utilized a combination of optical and cryo-electron microscopy techniques to analyze the architecture of TNTs in neuronal Cath.a-differentiated (CAD) and SH-SY5Y cells. Surprisingly, the study revealed that multiple individual TNTs (iTNTs) were bundled together through N-Cadherin-positive connections. These iTNTs contained various membrane-enclosed compartments and mitochondria, which appeared to be transported along parallel F-actin bundles with the assistance of molecular motors [42].

The speed of communication through TNTs varies depending on the type of material being transported. Electrical signal transduction via TNTs occurs within milliseconds, while the transport of organelles relies on motor proteins and is associated with rapid TNT polymerization. The spread of protein aggregates within TNTs is 2–8 times faster compared to other cellular components [43]. For instance, Tau aggregates move at a speed of 2.83 ± 1.99 µm/min in TNTs [19], and the transfer of viruses through TNTs exhibits an average speed of 0.08 ± 0.03 µm/s, which is approximately 2–5 times faster than the movement of the murine leukemia virus along filopodial connections [44]. Endosomes have a velocity of approximately 0.72 µm/min when transported by Rhes-mediated TNTs, which is approximately 150 times slower than the retrograde transportation of cargo observed in neurons. [2,23]. Actin, which is the major constituent of TNTs, represents a crucial focus for TNT imaging. It is hypothesized that F-actin-associated myosin motors play a role in facilitating the transportation of cellular components within TNTs [18,45,46]. Although microtubules have been detected in some TNTs, their exact role in TNTs remains unclear [47,48].

## 3. TNT-Like Structures in Development

Do TNTs have a role in brain development? According to the research findings, it has been observed that migrating neural crest cells (NCCs) establish and sustain ongoing communication with neighboring NCCs via both short and long filopodia during the process of neurogenesis. These connections play significant roles in guiding the directional movement of NCCs by allowing for cells to adjust their course based on the migration of neighboring cells [49,50]. This suggests that these contacts are crucial for coordinating the guidance of NCCs. Interestingly, TNTs have been found to be vital in early neuronal development by facilitating the transfer of calcium ions, which are necessary for regulating the proliferation, migration, and differentiation of neurons [51]. In the initial stages of maturation, developing neurons establish electrical coupling and exchange calcium signals with astrocytes through TNTs [39]. TNTs also enable the bidirectional transmission of electrical signals between NCC cells through the expression of connexin 43 [52]. TNTs can also bridge gaps during neural tube closure and contribute to the closure of the neural tube in the midbrain of mammalian embryos [53]. Thus, it is predicted that TNTs play roles in neuronal development and in the transmission of electrical signals as a critical mechanism in immature neuronal circuits [15]. They may contribute to the ongoing synchronization and coordination of migratory activity among NCCs within expanding tissues during embryogenesis.

## 4. TNTs in Glial Cells

Effective intercellular communication is essential between neurons and glial cells for the proper functioning and development of the brain [54]. TNTs have been reported to play a pivotal role in the brain’s detoxification process by removing toxic misfolded proteins via glial cells [16]. For example, TNTs have been shown to transport fibrillar α-synuclein (α-syn) from overloaded astrocytes to microglia, where it undergoes degradation [55]. Moreover, overloaded microglia can form TNTs, both in vitro and in vivo, to release excessive α-syn aggregates to neighboring microglia, which help in their clearance [55]. During such process, TNTs can prevent cell death and inflammation by transferring the mitochondria from naïve microglia to affected microglia [55]. Astrocytes and microglia can employ TNTs to facilitate the transfer of α-syn and mitochondria between astrocytes [7,56]. In another study, the authors demonstrate the occurrence of TNTs between human neuronal and microglial cells [7]. In this study, microglia showed a preference for transferring microglia to neuronal cells burdened with α-syn. These studies indicate that glial–TNT communication plays a critical role in decreasing the burden of accumulated protein aggregates and in promoting the mitochondria-mediated rescue mechanisms in neurodegenerative disease [7,56].

## 5. TNTs May Serve as Highways for Neurodegenerative Diseases in the Brain

TNTs are likely to have a crucial function in facilitating the transportation of different cargoes between brain cells. These cargoes include organelles, signaling molecules, pathogenic proteins, and microbes [7,14,16,24,57]. The transmission facilitated by TNT has the potential to influence both the physiological and pathological conditions of an organism. In particular, the intercellular transfer of various misfolded protein aggregates through TNTs holds significant implications for cellular communication and the development and progression of neurological disorders. 

Neurodegenerative diseases such as CJD, AD, PD, and HD share a common characteristic of protein aggregates that accumulate in the central nervous system, leading to selective neuronal cell death [58]. Recent research suggests that the progression of these diseases is associated with the spreading of these protein assemblies, which can influence the misfolding of other cellular proteins [17,19,58]. Specifically, there is evidence indicating that disease proteins, such as prion protein (PrP), amyloid beta (Aβ), tau, alpha-synuclein (α-syn), and huntingtin, can be transferred between cells through TNTs [7,25,58,59].

Studies suggest that prion protein can be transported through TNTs in two ways in CJD [60]. Firstly, due to the GPI-anchored nature of PrP, it can migrate along the surface of TNTs. Secondly, PrP transfer within TNTs may involve specific organelles or vesicles such as specific endosomal compartments [17,60,61,62]. Prions can also induce the formation of TNTs to facilitate their own intercellular transmission via endosomal vesicles [60]. Similarly, in rat hippocampal astrocytes and neurons, TNT-like structures are involved in the transfer of PrP and Aβ aggregates that are associated with AD [21,63]. Aβ is known to have toxic effects on neuronal cells. Cytotoxic Aβ aggregates can utilize TNTs as a means of spreading, thereby causing neurotoxicity [21,25]. Recent research suggests that oxidative stress and serum deprivation can unidirectionally transport extracellular Aβ through TNTs in rat hippocampal astrocytes and neurons. Interestingly, it was demonstrated that the transport speed of cytotoxic Aβ via TNTs is faster than that of cellular organelles [21,25], suggesting that TNTs can serve as superhighways for Aβ spreading and potentially disease progression. 

Like Aβ, the α-syn fibrils also spread through TNTs between various cells, including neurons, human neuronal precursor cells (hNPCs), and astrocytes, involving the Wnt/Ca^2+^ pathway [7,11,64,65]. Thus, cell-contact-dependent transfer appears to be a more efficient cell–cell transfer method of disease-relevant proteins. α-syn fibrils are shuttled predominantly inside lysosomal vesicles through TNTs involving ROS production [66,67]. Such transfer may also involve astrocyte intermediates, which further drive α-syn transmission to healthy astrocytes via TNTs, which could deliver mitochondria back to stressed astrocytes as two-way traffic [7,68]. Additionally, astrocytes can mediate the intercellular transfer of α-syn/MHC-II deposits through TNTs, indicating their involvement in the spreading of inflammation [69]. Thus, TNTs likely play multiple roles in the pathophysiological progression of PD.

In the case of HD, mutant huntingtin (mHTT) aggregates in neuronal cells and primary neurons increase the number of TNTs and promote their own spread through intercellular TNT-mediated communication [23]. Our recent research findings suggest that the Rhes protein, functioning as a multifunctional GTPase resembling SUMO E3 ligase, plays a vital role in the formation of TNTs and the facilitation of mHTT transportation between striatal medium spiny neurons (MSNs) and the cortex; the brain circuit is known to be highly vulnerable to degeneration in HD [2,4,22,23]. This transport of mHTT via Rhes is partly dependent on the SUMO posttranslational modification of mHTT [2,3,4,23,70]. An additional investigation is necessary to clarify the precise molecular mechanisms behind the propagation of mHTT through Rhes-TNTS and its possible contribution to the stereotypical degeneration observed in HD [71]. Similarly, misfolded α-synuclein aggregates, implicated in PD, are efficiently transferred through TNTs in primary neurons, CAD cells, SH-SY5Y cells, primary human brain pericytes derived from postmortem PD brains, and patient monocyte-derived microglia [11,55,65,66,72]. These aggregates often travel inside lysosomal vesicles and can seed soluble α-synuclein aggregation in the recipient cell cytosol [72]. 

The transfer of tau proteins across neuronal cells has been found to be facilitated by TNTs in astrocytes and neurons [19,21]. Both extracellular monomeric and fibrillar forms of Tau have been found to induce the formation of TNTs and promote their transfer between neuronal cells [19,24,73]. These observations support the idea that TNTs could serve as a means of spread for proteins that are involved across various neurodegenerative diseases. Therefore, further research is necessary to investigate the underlying mechanisms and pathways involved in the TNT-mediated mechanisms in neurodegenerative disease for exploring avenues for effective therapies.

## 6. Mitochondrial Transfer via TNTs

TNTs are a significant focus of current research and have been found to play a crucial role in the transfer of mitochondria, which are vital for cell survival [7,74,75]. Dysfunctional mitochondria have been associated with various neurodegenerative diseases such as AD and PD [7,76,77]. Factors such as reduced respiratory chain activity, accumulation of mtDNA mutations, and increased oxidative stress can all contribute to a decline in cellular bioenergetics [74,75,76,78,79,80,81]. Therefore, mitochondria have become prime targets for therapeutic interventions.

TNTs facilitate the transfer of mitochondria to a wide variety of cell types, leading to the restoration of mitochondrial function and the promotion of cellular survival [82,83]. Mesenchymal stem cells (MSCs) have been observed to transfer mitochondria and other beneficial biomaterials through TNTs, supporting the regeneration of tissues [84]. For instance, co-culturing MSCs with PC12 cells, a neuronal cell model, has shown to cause the transfer of mitochondria via TNTs, resulting in a reduction in apoptosis and restoration of mitochondrial membrane potential in damaged cells [84]. Inhibiting TNTs partially counteracted the positive effects of MSCs on injured PC12 cells, emphasizing the crucial role of TNTs in mitochondrial transfer. Similarly, MSCs have been found to transfer mitochondria to primary neurons through TNTs, enabling intercellular communication and the exchange of cytoplasmic materials [83,85,86]. These scientific inquiries have provided insights into the mechanisms that govern the intracellular transportation of mitochondria through TNTs, which presents new possibilities in cell therapy studies. TNTs are intricate structures that require careful examination and a better understanding of their mechanisms of action to optimize their utilization in cell therapy.

## 7. Molecules Involved in TNT Formation

The small GTPase family of proteins are recognized as critical modulators of TNT formation. The Rho family of small GTPases, particularly CDC42 and Rac1, has been extensively studied for their role in actin cytoskeleton rearrangement and the formation of filopodia and lamellipodia [14,87,88]. In immune cells and HeLa cells, CDC42 has been found to be associated with TNT formation, while Rac1 has not shown a significant effect. However, in neuronal CAD cells, inhibiting CDC42 and Rac1 increased the number of cells connected by TNTs, suggesting cell-specific mechanisms in TNT generation [88]. Additionally, the inhibition of the downstream effector of the Wiskott–Aldrich syndrome protein (WASP) and the WASP family verprolin-homologous 2 (WAVE2), Arp2/3, resulted in a decrease in TNT formation in macrophages but an increase in neuronal TNTs [14,88]. In fact, the same actin regulators were found to have opposing effects on filopodia and TNTs, such as the CDC42-VASP-IRSp53 network acting as a negative regulator of TNTs, while Eps8, which reduces filopodia formation, induces TNT formation and enhances cargo transfer [88,89]. These findings suggest a possible switch that determines the formation of either filopodia or TNTs. p53, a tumor suppressor, was shown to be necessary for the formation of TNTs [43]. When p53 function was disrupted either by using a dominant negative construct or its site-specific siRNAs, in both cases, TNT formation was hindered [43]. However, similar results were not observed in various other cell types, including murine bone-marrow-derived mesenchymal stem cells (MSC), PC12 cells, p53-null human osteosarcoma cells (SAOS-2), and OCI-AML3 (acute myeloid leukemia) cells [90]. These observations indicate a cell-specific pathway that regulates TNT formation and suggest that filopodia and TNTs use different mechanisms of formation. 

The Wingless-related integration site (Wnt) pathway, known for its involvement in actin cytoskeleton remodeling and filopodia formation, has also been investigated in relation to TNTs [64]. The activation of the Wnt/Ca^2+^ pathway has been shown to regulate the formation and stability of TNTs in neuronal cells and primary neurons by modulating actin and the β isoform of Ca^2+^/calmodulin-dependent protein kinase II [64]. Interestingly, while the Wnt pathway influences the formation of cytonemes (TNT-like structures in drosophila), it does not affect TNT formation, indicating differential regulation between the two structures [91].

MyosinX, an unconventional myosin motor protein, was found to play a critical role in inducing neuronal TNTs [18,45,92]. The motor and tail domains of MyoX, specifically the F2 lobe of the tail protein/ezrin/radixin/moesin (FERM) domain, are essential for neuronal TNT formation in neuronal cells. MyoX acts downstream of CDC42 and induces TNT formation independently of VASP, another inducer of dorsal filopodia formation. These findings suggest that neuronal TNTs may arise from a specific subset of MyoX-driven mechanisms of dorsal filopodia formation [14,91]. 

In neuronal CAD cells, it was demonstrated that the linear GTPase cascade involving Rab11a-Rab8a contributes to an increase in the number of TNTs [93]. This effect is dependent on the GTPase activities of Rab11a and Rab8a, which, along with their downstream effector VAMP3, a v-SNARE protein associated with endosomes, orchestrate TNT formation. Apparently, VAMP3 is not involved in inducing filopodia formation, suggesting its specificity in TNT formation [93]. Rab11a-Rab8a signaling is also implicated in TNT formation in cultured Schwann cells and dorsal root ganglion cells [94]. GTPase Rab35, a key regulator of membrane recycling [95], promotes the formation of TNTs and facilitates the transfer of vesicles through TNTs in neuronal CAD cells [96]. Along with its effector ACAP2, an Arf GAP (GTPase-activating protein), Rab35, forms a complex with together with GTPase Arf6-GDP to induce TNT formation by inactivating Arf6 [96].

**Figure 1:**
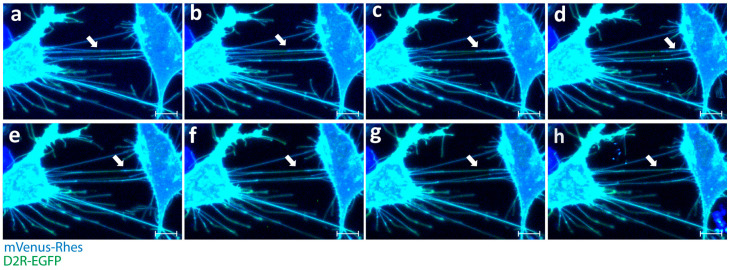
Rhes expression leads to induction of tunneling nanotubes and intercellular transport of D2R in striatal neuronal cells. (**a**–**h**) Confocal snapshots of live imaging (0–30 min) of mouse striatal cells transfected with mVenus-Rhes (blue) and D2R-EGFP. White arrowheads indicate Rhes tunnels connecting two cells. (Scale bar 10 μm.)

**Figure 2:**
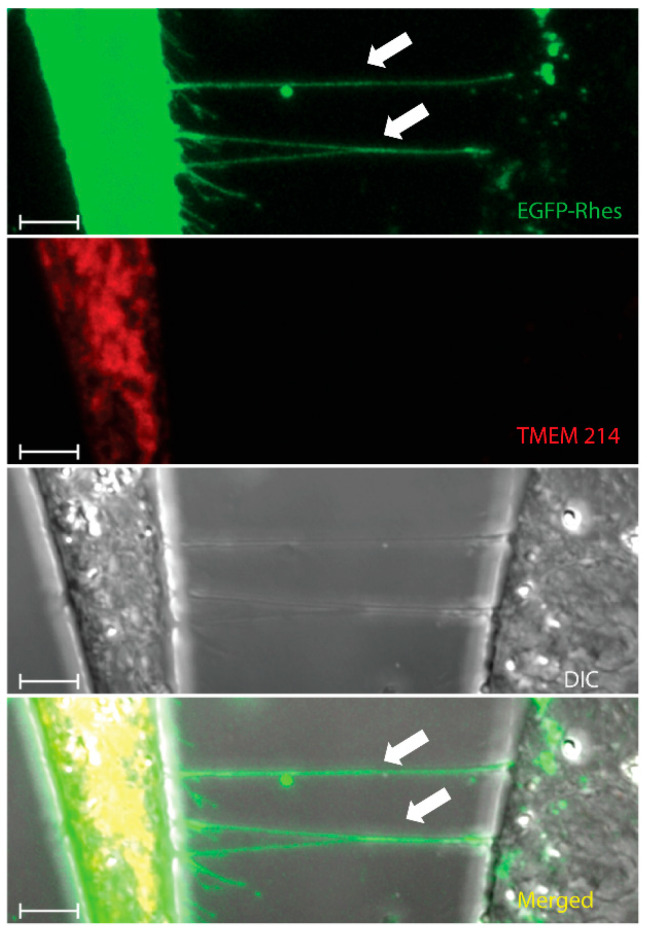
Rhes tunnels do not facilitate the intercellular transfer of TMEM214. Confocal snapshots of mouse striatal cells transfected with EGFP-Rhes (Green) and mCherry-TMEM214 (Red). White arrowheads indicate Rhes tunnels between two cells. (Scale bar 10 μm.). Green = EGFP-Rhes, Red = TMEM 214.

**Figure 3:**
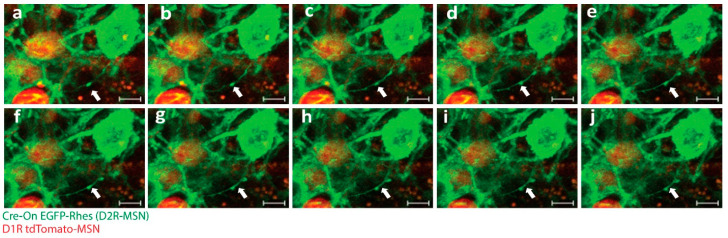
Rhes facilitates TNT-like protrusions in mice brain. (**a**–**j**) Confocal snapshots of time-lapse live imaging of mice brain slices. White arrowhead indicates EGFP-Rhes-positive TNT-like protrusions connecting D1R tdTomato-MSN (red) to D2RCre-MSN (green). (Scale bar 5 μm.)

As discussed above, we identified the involvement of Rhes in the formation of TNTs [2,3,22,35,97]. While Rhes is self-transported via TNTs (Figure 1 and Figure 2; Video S1), it also facilitates the transport of the poly-Q expanded mHTT protein that is associated with HD [23]. The Rhes effect on mHTT transport is specific, as normal huntingtin protein, mTOR, or wild-type Tau proteins are not transferred by Rhes-TNTs [23]. Specific regions within the Rhes protein, including serine 33, a C-terminal SUMO E3-like domain and membrane-binding CAAX motif, play critical roles in TNT formation and Rhes and mHTT transmission [23]. As shown before [22], Rhes-TNTs are involved in the intercellular transport of only specific receptor cargoes, for example, receptors like histamine-3, dopamine receptor D1 (D1R), and dopamine receptor D2 (D2R), from one cell to another, but not TMEM214, an endoplasmic reticulum-associated transmembrane protein (Figure 2 and Figure 3) [22]. This suggests that Rhes mediates the transport of selective cargoes through TNTs between cells.

S100A4 is a small extracellular calcium-binding protein that regulates the direction of emerging TNTs towards their target cells in neurons and astrocytes [98]. Through a combination of microscopic and proteomic analyses, it was discovered that S100A4, along with its receptor, RAGE (receptor for advanced glycation end products), plays a crucial role in directing the formation of TNTs towards their intended recipient cells. In TNT-forming cells, p53 activation leads to the activation of the pro-apoptotic protease caspase-3 that cleaves S100A4, reducing its cellular concentration within the TNT-forming cell [98]. This reduction appears to help maintain an increasing concentration gradient of S100A4 towards the target cell, facilitating TNT formation. It remains uncertain whether the extracellular concentration of S100A4 itself can modulate caspase-3 activation levels in donor or acceptor cells. However, it was observed that S100A4 and its receptor can guide the directional elongation of TNTs between homotypic astrocyte–astrocyte connections or heterotypic astrocyte-HEK293 connections [98]. These studies imply that cell-type-specific mechanisms operate in the formation of TNTs.

## 8. Challenges Involved in Researching TNTs in the Brain

Studying TNTs in neurons and in the brain presents a unique set of challenges due to the complex nature of these structures and the dynamic environment in which they exist. One of the primary challenges in studying TNTs in neurons and the brain is their small size and fragile nature. TNTs are thin, delicate structures that are difficult to isolate and visualize. Nevertheless, advanced imaging techniques, live-cell imaging, and electron microscopy are routinely used to visualize TNTs, but these techniques require specialized equipment and expertise [34,42]. In addition, the fragility of TNTs makes them difficult to manipulate and study in vitro, as they are easily damaged during cell isolation and culture. Another major challenge in studying TNTs in neurons and the brain is their dynamic nature. TNTs are highly dynamic structures that can rapidly form and disappear in response to various stimuli [6,99,100,101]. As a result, it is challenging to study the static nature of TNT formation and function in vivo between cell types. Transgenic mice with cell-type-specific markers are necessary to visualize and follow TNT-like structures between cell types in a 3D brain slice culture. As an example, we successfully produced transgenic mice named D1-MSN^Cre^ and D2-MSN^mcherry^. The utilization of this technique enabled the observation and tracking of “cre-on” EGFP-Rhes-mediated creation of TNT-like structures between D1-MSN and D2-MSN in an ex vivo setting (Figure 3; Video S2). Moreover, conducting research with the objective of identifying a potential distinct marker of TNTs would enhance the ability to detect and characterize TNTs, distinguishing them from other types of plasma membrane protrusions in both in vivo and in vitro specimens [22]. 

## 9. Future Prospects of TNTs in Brain Research

Neuroplasticity is the brain’s ability to change and adapt in response to new experiences, learning, and environmental stimuli [102]. As indicated above, TNTs have been shown to facilitate the transfer of cytoplasmic material, including proteins and genetic material, between cells [103,104]. Thus, they have the potential to play critical roles in neuroplasticity by facilitating the transfer of signaling molecules between neurons. TNTs might also be involved in synaptic plasticity, which is the process by which synapses between neurons are strengthened or weakened based on the activity of those neurons. Understanding the role of TNTs in synaptic plasticity could lead to the development of new treatments for largely untreated neurological disorders, such as autism and schizophrenia, which are characterized by abnormal synaptic plasticity.

As described above, TNTs play a crucial role in the spread of misfolded proteins in neurodegenerative diseases [3,16,24,58,59]. Therefore, the development of strategies that block the formation and/or specific transfer function of TNTs may slow or halt the progression of neurodegenerative disorders. Additionally, TNTs may be potential targets for drug delivery to the brain. The ability of TNTs to transfer cytoplasmic material between cells could be harnessed to deliver therapeutic molecules, such as RNA interference, directly to neurons that are affected by diseases. More recently, syncytin-mediated TNT-like structures were linked to the efficient transfer of the Cas9/gRNA complex between cultured cells, which suggests the possibility of directly delivering gene-altering cargoes via TNTs [105]. Thus, further studies may help to identify TNT as a means of delivering gene therapy cargoes to specific cell types in in vivo and ex vivo therapies.

TNTs have also been shown to play a role in neuron regeneration [94,106], which has the potential to be harnessed for the treatment of spinal cord injury and other neurological disorders. Memory formation and consolidation involve complex mechanisms that are still not fully understood. TNTs have been shown to play a role in the transfer of mitochondria, which are critical for energy production and memory [107]. It would be interesting to study if TNT-mediated transfer may be essential for synaptic plasticity and the formation of new memories. 

TNTs also hold a promising future for the intercellular transfer of nanomedicine to cure neurodegenerative diseases. TNTs have been reported to transfer quantum dot nanoparticles (NPs) in rat cardiac myoblast cells [108], suggesting that TNTs could serve as delivery mechanisms for nanomedicines in HD [109] and AD [110]. For example, one can develop a strategy to deliver KVLFF-encapsulated NPs via TNTs to diminish amyloid β aggregates in a cell-type-dependent manner [110]. Similarly, TNTs can also be employed to deliver cholesterol-encapsulated NPs in HD [109]. Future in-depth studies would be crucial to identify cargo delivery mechanisms involved in the transport of NPs via TNTs that could bypass the extracellular matrix. 

## 10. Conclusions

Besides chemical and electrical synapses, it is highly probable that TNTs possess significant potential to fundamentally transform our comprehension of the structure and functioning of the brain, as well as offer novel therapeutic possibilities for neurological illnesses. The investigation of TNTs is currently at its nascent phase, and there remains a considerable amount of knowledge to be acquired regarding their underlying mechanisms, regulatory processes, and prospective utilizations. Although recent developments in experimental techniques have revealed many new findings, there are still several unanswered questions. For instance, the precise biophysical characteristics of these structures and the mechanisms involved in cargo transportation are not yet fully understood. Moreover, it is unclear what triggers the formation of different types of TNTs and how the donor and recipient cells determine which cargoes to transfer. Furthermore, the number of cells connected through TNTs at any given moment and the extent of their biological impact in various tissues and diseases remain unknown. Conducting more research in this area is critical to comprehend the role of TNTs in maintaining tissue homeostasis and promoting tissue regeneration, as well as their potential contribution to brain illnesses such as cancer and neurodegenerative disorders.

## Data Availability

All data are contained within this manuscript.

## Supplementary Materials

The following supporting information can be downloaded at https://www.mdpi.com/article/10.3390/biology12101288/s1, Video S1: Rhes promotes induction of tunneling nanotubes and intercellular transport of D2R in mouse striatal neuronal cells. Mouse striatal cells are transfected with mVenus-Rhes (blue) and D2R-EGFP(green). (Scale bar 10 μm); Video S2: Rhes promotes biogenesis of TNT-like structures in brain. TNT-like protrusions connecting D1R tdTomato-MSN (red) to D2RCre-MSN (green). (Scale bar 5 μm).
